# Geochemical characteristics, hazards impact assessment and radiogenic heat production of the alkaline rocks

**DOI:** 10.1038/s41598-024-59627-x

**Published:** 2024-04-20

**Authors:** Essam Sidique, Mervat A. Elhaddad, Mabrouk Sami, Ioan V. Sanislav, Fahad Alshehri, Mohamed S. Ahmed, Hassan Abbas

**Affiliations:** 1 Department of Physics, Faculty of Science, New Valley University, El-Kharga, 72511 Egypt; 2 Department of Geology, Faculty of Science, Assiut University, Assiut, 71516 Egypt; 3 Geosciences Department, College of Science, United Arab Emirates University, 15551 Al Ain, United Arab Emirates; 4 Geology Department, Faculty of Science, Minia University, El-Minia, 61519 Egypt; 5 Economic Geology Research Centre (EGRU), College of Science and Engineering, James Cook University, Townsville, QLD 4811 Australia; 6 Abdullah Alrushaid Chair for Earth Science Remote Sensing Research, Geology and Geophysics Department, College of Science, King Saud University, 11451 Riyadh, Saudi Arabia

**Keywords:** Alkaline rocks, Natural radionuclides, REEs Geochemistry, HPGe detector, Radiological hazards, Environmental sciences, Natural hazards

## Abstract

This study primarily investigates the natural radioactivity level in alkaline rocks collected from the Wadi El-Dib ring complex (WDRC) in North Eastern Desert of Egypt, and assesses potential health risks associated with their use as decorative building materials. The work was accomplished using a high-purity germanium detector as well as ICP-MS and ICP-AES techniques. The WDRC composed essentially of trachyte, quartz syenite, granite and syenite. Geochemically, these rocks contain high SiO_2_ and alkalis with metaluminous to slightly peraluminous features. All rocks contain high concentrations of rare earth elements (∑REEs = 109–1075 ppm), with clear enrichment in light REEs compared to heavy REEs [(La/Yb)_N_ = 8.3–25.3. Radiometrically, the concentrations of the natural radioisotopes (^238^U, ^232^Th, and ^40^K) in the studied rock types surpassed the worldwide average values assigned for building materials by UNSCEAR. This elevation of the radioisotope concentration values is due to the presence of supplement minerals such as monazite, zircon, allanite, and rutile. Granites exhibit the highest mean concentrations of ^238^U (av. 164.24 ± 14.76 Bq/kg) and ^232^Th (av. 214.37 ± 23.33 Bq/kg), while trachytes demonstrate the highest ^40^K (av. 1352.56 ± 65.56 Bq/kg) concentrations. In contrast, syenites exhibite the lowest mean concentrations for ^238^U (av. 54.51 ± 6.81 Bq/kg) and ^232^Th (av. 56.76 ± 6.25 Bq/kg), while quartz syenites display the lowest mean concentration of ^40^K (av. 1144.78 ± 96.19 Bq/kg). The radiogenic heat production (RHP) associated with U, Th, and K range between 1.41 to 9.33 μW/m^3^, exceeding the typical crustal mean value of 0.8 to 1.2 μW/m^3^. The radiological parameters and indices evaluating risks of the outdoor and indoor radiation doses due to the investigated rocks were assessed. The results indicated that these rocks meet globally accepted values and safety standards (approved by UNSCEAR, ICRP, and EC) for surface building materials, as well as they underscore the importance of adhering to safety protocols to safeguard workers from radiation exposure within the WDRC area. Ultimately, the data herein provide a valuable database for assessing the compatibility of geochemical data and natural radioactivity level in WDRC rocks. Additionally, it reveals that from the radiological perspective, the investigated rocks are considered safe for use as decorative construction materials.

## Introduction

The primary source of natural radioactivity in rocks emanates from the presence of radionuclides (e.g., ^238^U, ^232^Th, and ^40^K), which intricately linked to their mineralogical composition^1,2^. Amidst the growing public apprehension regarding radiation exposure, there has been a surge in studies focusing on high-background natural radiation areas. These investigations particularly center around naturally occurring radioactive materials (NORMs)^3,4^. In fact, mountainous regions containing alkaline rocks and other igneous varieties are known for emitting elevated levels of natural radiation due to the presence of such NORMs. As a result, people living or working in these areas are exposed to increased levels of radiation, which pose health risks if proper safety measures are not followed. However, these levels of natural radiation can vary greatly depending on rock mineralogical and geochemical composition and tectonic settings^5^. Natural radiation in alkaline rocks primarily arises from the presence of specific radioactive elements like U, Th, and K. These radioactive elements decay over time, emitting radiation^6,7^. The specific mineral composition of granitic and alkaline rocks, which often includes minerals like zircon, monazite, allanite, thorite, apatite and uraninite, can also influence their radioactivity due to the concentration of radioactive elements within these minerals^8^.

Radioactive decay of the terrestrial radioisotopes (^238^U, ^232^Th, and ^40^K) is the primary cause of radiogenic heat production (RHP) in rocks^9^. The heat generated due to the decomposition of these radioisotopes in rocks represents an important element in geothermal research, particularly in interpreting the Earth's continental heat flow. It is responsible for a significant portion (about 98%) of the heat flow^10^.

Human activities can contribute to individuals' exposure to natural radiation^3,11^. One of the most important human activities associated with natural resource exploitation is the use of rocks as building materials. Exposure to radiation from these materials can occur through the decay of the radioactive elements over a long period of time and cause a risk to human health, depending on the level and duration of exposure^12,13^. Therefore, it is important to consider examining rocks for radioactivity before using them as building materials.

The radioactive elements and rare earth elements (REEs) are mainly sourced from resources such as phosphorites^2^; ironstone^14^; black shale^15^, rare metal granites^8^; pegmatites^16^, carbonatites and alkaline-rich rocks^17^ among others. Notable areas for REEs include China (Bayan Obo deposit)^18^, Australia (Mount Weld deposit)^19^, and Brazil (Catalão deposit)^20^. Significant U and Th deposits can be found in regions like Canada (Athabasca Basin)^21^, Kazakhstan (Inkai deposit)^22^ and Niger (Arlit deposit)^23^. Magmatic rocks can be important sources of radioactive elements, conventionally, trapped within rare metal and radioactive minerals (e.g., zircon, thorite, uraninite, and monazite) in these rocks^24,25^. Uranium and thorium, natural radioelements, are lithophile elements found extensively throughout crustal rocks, with a notable preference for accumulating in silicic magmatic rocks over their intermediate, mafic, and ultramafic counterparts. Thorium is more abundant than uranium in crustal rocks due to its lower susceptibility to mobilization within the supergene environment^26^. In Egypt, magmatic rocks, notably alkaline varieties like granite and syenite, have extensively used as building materials. Alkaline rocks, in general, are known for their elevated U and Th levels, due to the distinct characteristics of the originating magma and its related tectonic setting. Typically, rocks formed within the Earth's crust tend to contain higher concentrations of radioelements compared to those originating from the mantle. This disparity arises from processes like partial melting and fractionated crystallization, which concentrate these elements in the liquid phase of silica-rich magma^8^.

Many magmatic rocks have an attractive appearance not only in Egypt but also worldwide. Egypt is a leading producer of ornamental stones, underscoring the importance of assessing their radiological effect on health. As the population grows, so does the demand for ornamental stones. The study area, Wadi El-Dib Ring Complex (WDRC) contain a variety of extrusive (trachyte) and intrusive (quartz syenite, granite, and syenite) alkaline rocks which haven't been previously covered for their health impacts. The main target of this study is: (1) discussing the petrological and geochemical characteristics of WDRC rocks; (2) evaluation of the RHP in these rocks, as they could have potential for geothermal energy and (3) assessment of their radiological hazards, which provide important insights into the public's exposure to radiation.

### Geological background and petrography

**Figure 1:**
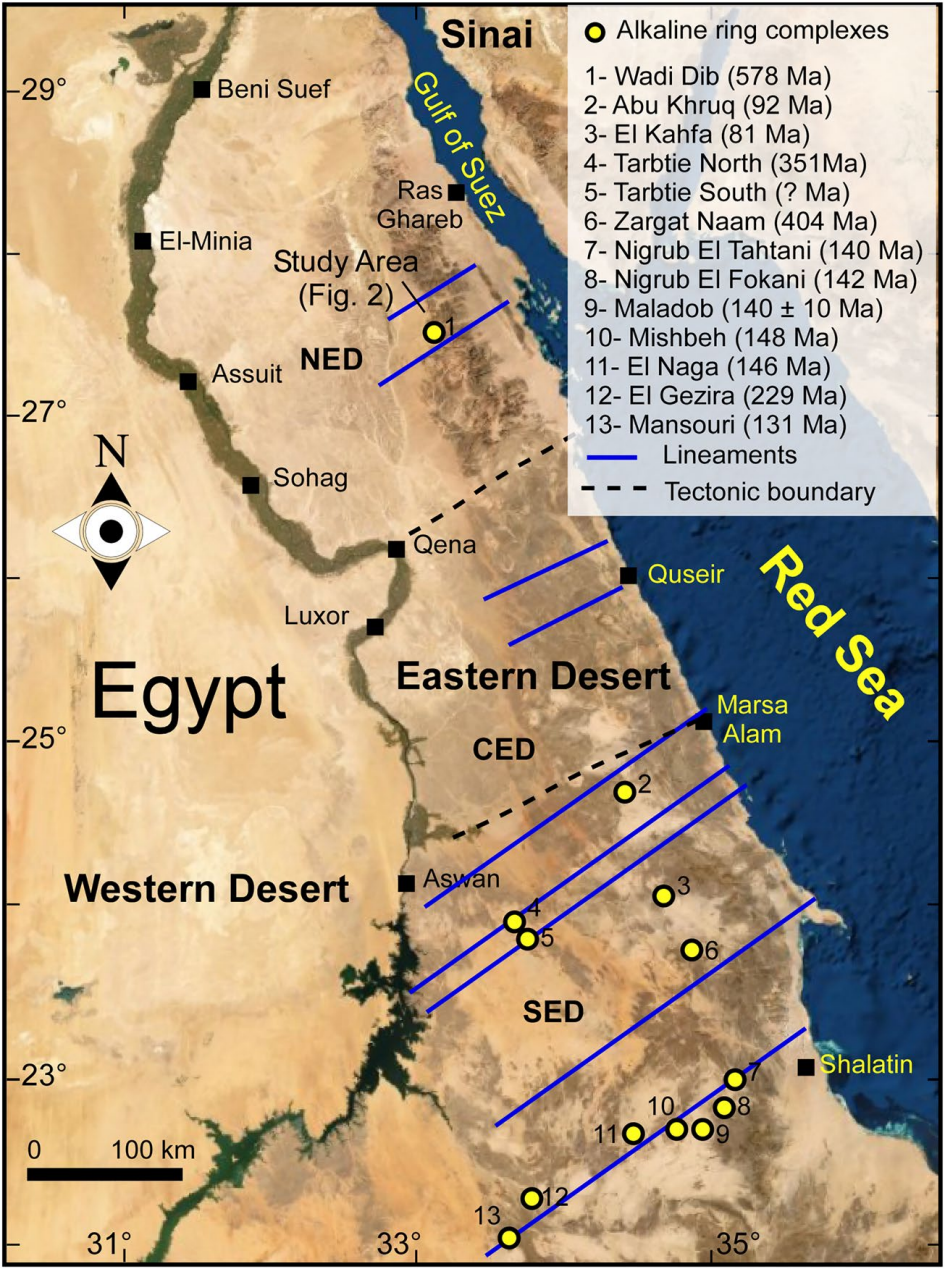
Key map illustrating the spatial distribution of alkaline ring complexes across the Eastern Desert of Egypt (after Abdel-Karim, et al.^31^).

**Figure 2:**
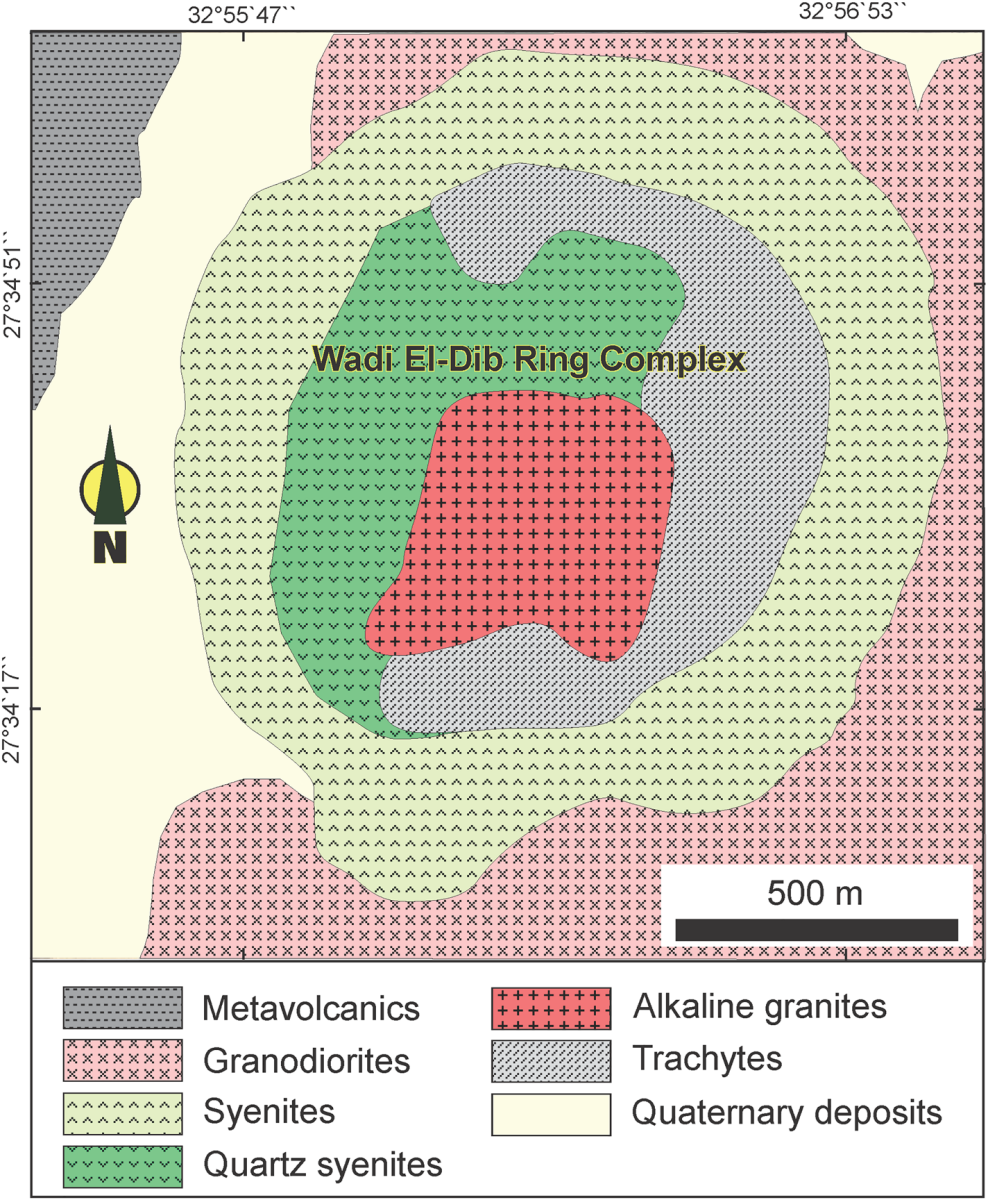
Geological map of WDRC, North Eastern Desert of Egypt. “This map was created by using Corel Draw software v. CorelDRAW Standard 2021; https://www.coreldraw.com/en/product/coreldraw/standard/).

The Nubian Shield, encompassing Egypt, Sudan, and Ethiopia, boast a registry of over 130 alkaline ring complexes. Their emplacement spans a temporal range from the Neoproterozoic (~ 650 Ma) to the Oligocene (~ 25 Ma)^27^. Notably, these complexes demonstrate close spatial association with significant deep-seated fault zones^28^. The alkaline activity and occurrence of ring complexes in the Egyptian Southern Eastern Desert is linked generally to the tectonic and magmatic activities. They emplaced between the closing stages of subduction-related calc-alkaline magmatic activity at the end of the Pan-African orogeny (650–500 Ma)^29^ and the opening of the Red Sea (35–25 Ma)^30^. The ring complexes in Egypt, situated in the Southern Eastern Desert, encompass locales like Abu Khuruq, Mishbeh, El- Naga, El-Gezira, Tarbtie (N and S), Nigrub, Meshbeh, Maladob and Mansouri^31^. Among these, the oldest Wadi El-Dib Ring Complex (WDRC) is located in the north Eastern Desert (Fig. 1)^32^. It represents the oldest ring complex (~ 578 ± 16 Ma; Rb–Sr isotopes of syenite, trachyte and granites) in the Egyptian basement complex^32^. Geologically**,** WDRC is an intrusive circular body (2 km in diameter) that is oval or arcuate in plan-with steep contacts and consists of ring sheets (Fig. 2). The outer rings comprise syenites and pegmatitic syenites, while the inner sections host quartz syenites surrounding a core of fine-grained alkali-feldspar granites. The post intrusive mafic (basaltic) and felsic (rhyolitic) dikes cut all the WDRC rocks with NNW-trending. The mafic dikes are composed of dark colored minerals like mica, amphibole and pyroxenes, while the felsic dikes are light colored composed of quartz and feldspars. The ring emplacement took place-around ring fractures at a subvolcanic level i.e., the magma was intruded around circular fractures forming the ring structure^32^. The WDRC was emplaced at the intersection of two ENE and ESE trending faults^32^, in the late-to post-Pan-African of the Najd fault system^33^. The contacts between the ring sheets slopen (almost vertically) from the margin towards the intermediate trachytic unit, while the inner part showed contacts steeply dipping towards the ring center.

**Figure 3:**
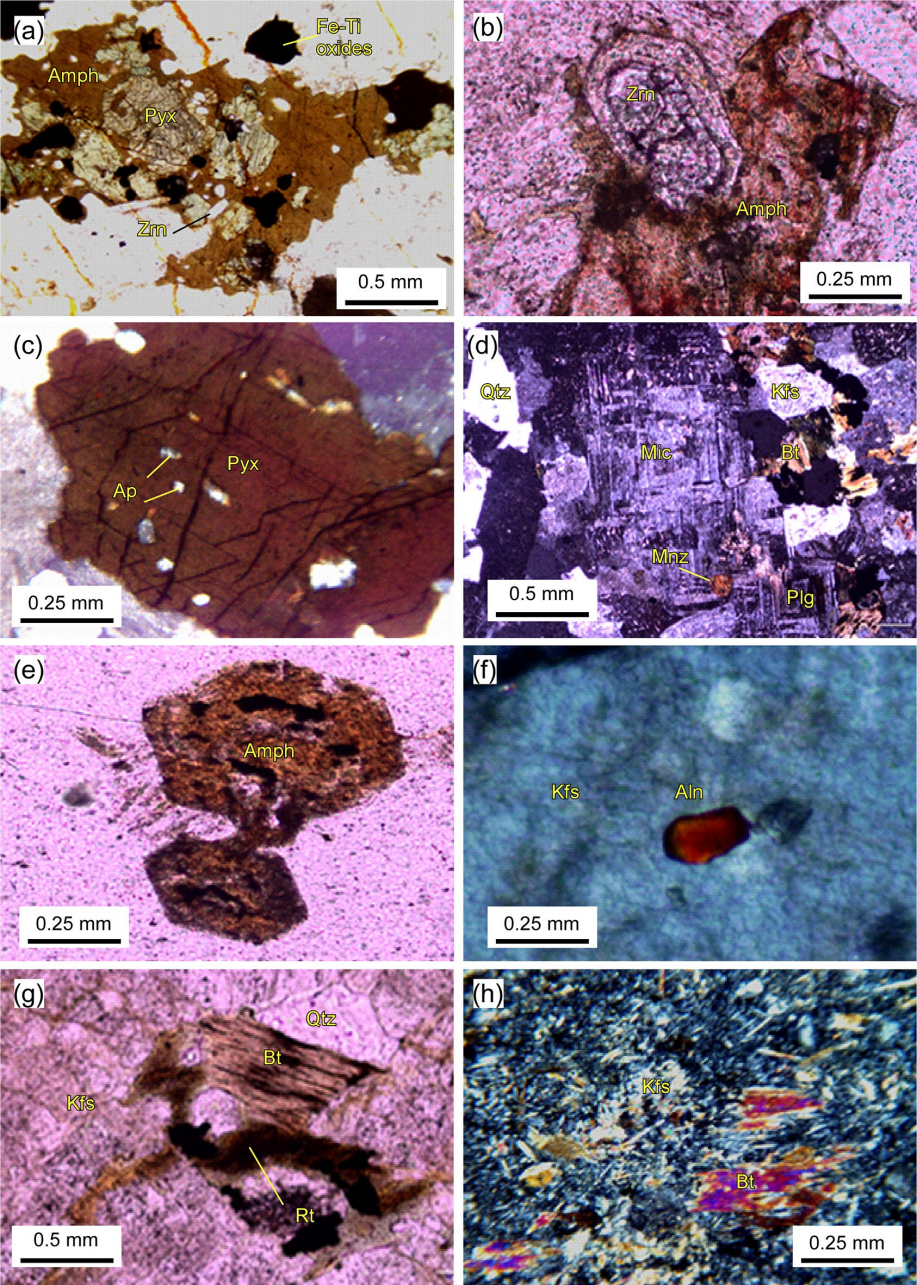
Detailed photomicrographs of WDRC rocks: (**a**) pyroxene (Pyx) crystal encompassed by amphibole (Amph) (syenite, PPL); (**b**) occurrence of a well-formed, euhedral zircon (Zrn) crystal (syenite, PPL); (**c**) anhedral pyroxene crystal hosted apatite (Ap) and other accessory phases (syenite, PPL); (**d**) the hypidiomorphic texture of granite, with monazite (Mnz) occurs between microcline (Mic) and plagioclase (Plg) (granite, XPL); (**e**) euhedral alkaline amphibole crystals within a granite (PPL); (**f**) occurrence of allanite (Aln) hosted by k-feldspar (Kfs) in granite (PPL); (**g**) Rutile (Rt) inclusions within biotite (Bt) and quartz (Qtz) (PPL); and h) microphenocrysts within the younger trachyte groundmass (XPL).

The rock samples collected from WDRC were classified as syenites, quartz syenites, trachytes and granite, depending on their mineralogical composition and textures. The syenites are medium grained with hypidiomorphic texture. In hand specimen the color varies from light grey to reddish. Petrographically, it is composed of K-feldspar, plagioclase, alkali-amphibole and pyroxene. The main accessory minerals are zircon, allanite and apatite. K-feldspar crystals make up ~ 65% (vol.%), the crystals are subhedral to anhedral, commonly display Carlsbad twinning. Subhedral to anhedral plagioclase crystals (albite) form ~ 10% (vol.%). The alkali-pyroxenes (Fig. 3a) comprise ~ 18% (vol.%), occur interstitial to quartz and K-feldspars. They were partially replaced by amphibole as well as by iron oxides. Inclusions of zircon and allanite are common. Opaques and accessory minerals form about 2–3% (vol.%), associated with mafic minerals. Allanite occurs as reddish brown to dark brown crystals, distributed over the minerals. Zircon (Fig. 3b) occurs as prisms enclosed within pyroxene and potash feldspar. Apatite is rare, when present enclosed within feldspars as long, thin, slender crystals. The quartz syenites (Fig. 3c) is coarse grained having almost the same mineral composition of the syenite.

The granites are whitish, inequigranular and coarse-grained rocks consist of potash feldspar, quartz, plagioclase, amphibole, biotite and Fe-Ti oxides. The predominant mineral is the K-feldspar (30 vol.%), rarely exhibits simple twinning, consists of orthoclase and microcline (Fig. 3d). Plagioclase (35 vol.%) appears as euhedral megaphenocrysts and microphenocrysts. The rims of phenocrysts are usually altered into sericite. Quartz (25 vol.%) crystallized as interstitial anhedral microcrystals. Biotite (5–7 vol.%) appears as oxidized dark brown platelets. Biotite crystals always enclose apatite and zircon, the microphenocrysts are sometimes included into the alkali feldspar. Amphiboles (1–2 vol.%) are euhedral crystals (Fig. 3e) of various shapes and sizes often altered into opaque. The accessory mineral phases (~ 1 vol. %) are made up of zircon, allanite, sphene, rutile and apatite which are usually included in the major mineral phases (Fig. 3f,g). The trachyte shows a variable texture feature where some samples have fine-grained groundmass with equigranular crystals of alkali feldspar, biotite and few undefinable microphenocrysts (Fig. 3h), while others contain microcrystals of alkali feldspar and quartz.

## Investigative methodologies

### Sampling and analytical methods

For this study, a total of twenty-four fresh rock samples representing all rock types of the WDRC were examined (Fig. 2). The samples were labeled and stored in burlap sacks before being transported to the laboratories. Thin sections were prepared for the petrographic study. For the preparation of the radioactivity measurements and whole rock analyses, each sample was divided into two parts: the first part for radiometric investigation using the high-purity germanium (HPGe) detector, while the second one was prepared for the inductively coupled plasma mass spectrometry (ICP-MS) combined with the atomic emission spectrometry (ICP-AES) analyses. Before radiometric measurements, each sample underwent individual grinding and sieving (200 μm mesh). After oven-drying at 105 °C for 5 h to remove moisture, samples (600–850 g) were weighed. They were then placed in plastic cylindrical containers (48 mm radius, 82 mm height, 0.5 mm thickness), left for over 4 weeks to attain secular equilibrium.

### Whole-rock geochemistry

The major, trace, and rare earth elements REE of the samples under consideration were analyzed to confirm the gamma spectrometric analysis and to characterize the WDRC rocks from a chemical standpoint. The whole-rock analyses were carried out in the OMAC International Certified Laboratory (Loughrea, Ireland). In each analytical procedure, 0.2 g of each sample was mixed well with 0.90 g of lithium metaborate before being melted in a furnace at 1000 °C. The molten material was cooled then dissolved in 100 ml of 4% HNO_3_ (nitric acid) or 2% HCl_3_ (hydrochloric acid) solution. The resulting solution was examined for the major elements using ICP-AES (ALS code ME-ICP06) and the REEs using ICP-MS (ALS code ME-MS81). For the major oxides, U and Th (trace elements), and the REE elements, the detection limits were 0.01%, 0.05 ppm, and 0.01–0.5 ppm, respectively. The findings were adjusted to account for spectrum inter-element interferences. Additional extensive information on the OMAC lab's analytical methodologies and preparations is found at www.alsglobal.com, accessed on September 15, 2023. The yielded Th and U elemental concentrations in ppm (ICP-MS analysis) have been converted to the activity concentrations in Bq/kg of ^232^Th and ^238^U, as well as the yielded K concentration in percent (%) to ^40^K in Bq/kg^34,35^, where 1% of K = 309.7 Bq/kg, 1ppm of U = 12.35 Bq/kg and 1ppm of Th = 4.07 Bq/kg.

### Gamma spectrometric analysis

The radionuclide content of samples was determined using a coaxial HPGe detector (Canberra, GR4020 model) with an extended range of energy (10 keV to 3 MeV), a relative efficiency of 40%, and a resolution of 2 keV for the Co-60 gamma line (1.332 MeV). A cylindrical lead shield (Model 747E, Canberra) was used to secure the detector, averting more than 98% of the background noise from reaching it. For data acquisition, signals are routed through an amplifier (Canberra, Model 2002CSL) to a Canberra DSA-1000 16k channel multichannel analyzer using two analog-to-digital converters. The GENIE-2000 software was used to acquire and analyze the gamma spectra. After subtracting the background peaks, the software computes the isotope's activity concentration from the outstanding gamma peaks.

**Figure 4:**
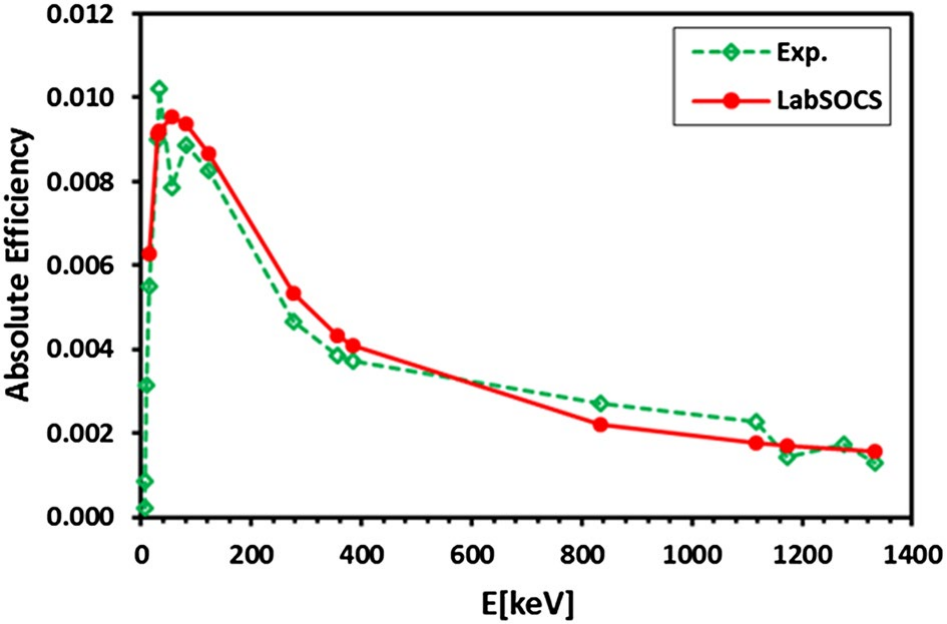
Absolute efficiency curves for experimental measurements (Exp.) and Laboratory Source Calibration Software (LabSOCS) predictions across energy levels (E) in keV.

Prior to the measurement, the detector's energy and efficiency were calibrated using LabSOCS (Laboratory Sourceless Calibration Software). The LabSOCS software can be accessed through the Geometry Composer which can be launched from the Genie 2000 software. The latter comprises the detector's characterization files set up and established through the system manufacturer's basic calibration experiments using gamma ray (Ba-133, Co-60, Cs-137, Mn-54, Na-22, and Zn-65) point sources. During the execution of the calibration using LabSOCS, the sample-to-detector geometry, the sample's composition, density, dimensions, and characteristics of the beaker containing the sample were all taken into consideration. To verify the accuracy of the efficiency values provided by LabSOCS, our laboratory conducted a series of experiments using a collection of standard point sources (Ba-133, Co-60, Co-57, Mn-54, Na-22, and Zn-65) had been positioned at varying distances (0–15cm) from the detector's end-cap. By applying Eq. (1)^36,37^, the absolute full-energy peak efficiency (ε) was evaluated.

1 $$\varepsilon =\frac{N}{A\cdot t\cdot I}$$

where *N*, *A*, *t*, and *I* are the net area count, activity in Bq, live time in seconds, and branching ratio fraction, respectively. It has been found that the efficiency values generated by LabSOCS correspond closely to those determined through our experimental investigations, as illustrated in Fig. 4.

As for the investigated rock samples, the counting time of the measurements (forming their spectrum) was at least 10 h. For counting under the same conditions, an empty cylindrical beaker was put on the detector to obtain the background spectrum before each measurement. The ^238^U activity in the samples was determined via the gamma ray’s lines with energies of 609.31, 1120.28, and 1764.49 keV resulting from ^214^Bi decay and 295.22 and 351.93 keV due to ^214^Pb disintegration. Through the use of gamma ray’s lines with energies of 338.32, 911.20, and 968.97 keV from ^228^Ac decay, 583.19 and 2614.53 keV from ^208^Tl disintegration, and 238.63 keV from ^212^Pb, the ^232^Th activity in the samples was identified. As for ^40^K, only the gamma ray line of 1460.86 keV originating from its own single decay was used to specify its activity. As per Eq. (2) below, the activity concentration (AC) of the aforementioned radionuclides in every sample was calculated from the corresponding gamma lines of energy E while taking into account the mass of the sample (M_s_), net peak count (N_c,E_) at energy E, gamma decay transition probabilities I_γ,E_, and detector efficiencies (ε_E_), as reported by Sidique, et al.^38^ and El-Gamal, et al.^34^.

2 $$AC\left[Bq/kg\right]=\frac{{N}_{c,E}}{{I}_{\gamma ,E}\cdot {\varepsilon }_{E}{\cdot M}_{s}}$$

The detection limit (LD) of the spectrometer, indicating its ability to detect gamma rays despite natural interference, and the minimum detectable activity (MDA) were calculated using Eqs. (3a) and (3b), respectively, as follows^31,32^**:**

3a $$DL=2.71+4.66\sqrt{{NB}_{c,E}}$$

3b $$MDA\left[Bq/kg\right]=\frac{DL}{{I}_{\gamma ,E}\cdot {\varepsilon }_{E}{\cdot M}_{s}}$$

where *NB*_*C,E*_ is the background count rate at gamma lines of energy *E*^31,32^.

## Results and discussion

### Geochemical characteristics

**Figure 5:**
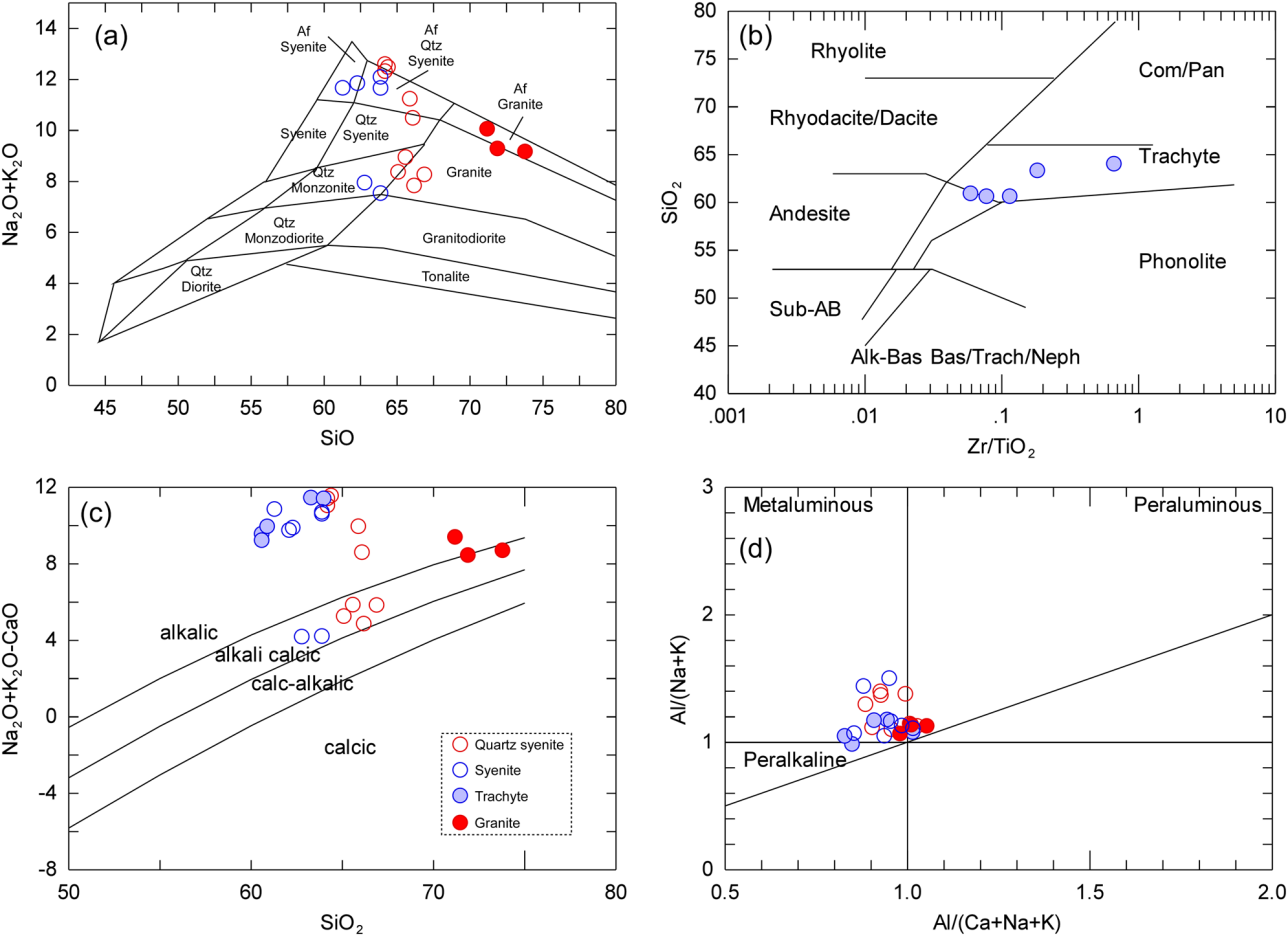
(**a**) plot of SiO_2_ vs. Na_2_O + K_2_O showing the classification of the studied plutonic rocks of WDRC^61^; (**b**) SiO_2_ vs. Zr/TiO_2_ diagram shows the classification of the studied volcanic rocks of WDRC^62^; (**c**) Na_2_O + K_2_O-CaO vs. SiO_2_ relation indicate the alkali calcic and alkalic nature of samples^39^; and Al/(Na + K) vs. Al/(Ca + Na + K) binary relation displaying the metaluminous to slightly peraluminous geochemical characters of the studied rocks^39^.

The major and trace elements abundances of WDRC samples are listed in the Supplementary Table S1. The samples contain high concentration of SiO_2_ (73.80–60.60 wt%), Al_2_O_3_ (19.45–13.65 wt%), and total alkalis (Na_2_O + K_2_O = 7.50–12.99 wt%) with variable concentration of CaO (0.45–3.74 wt%) and Fe_2_O_3_ (2.15–5.85 wt%). The rocks of WDRC have been classified using the SiO_2_ vs. K_2_O + Na_2_O and Zr/TiO_2_ vs. SiO_2_ and classification diagrams (Fig. 5a,b), where the plutonic samples fill the fields of syenite and granites (Fig. 5a) and the volcanic samples occupy the trachyte field (Fig. 5b), consistent with the field and petrographic investigation. The alkaline affinity of the studied rocks is supported by their high alkalis content and by the diagram of Frost, et al.^39^ (Fig. 5c), where all samples are clustered in the alkali and alkali-calcic fields. Using the binary relation between the A/NK vs. A/CNK (Fig. 5d), all WDRC rocks are further characterized by their metaluminous to slightly peraluminous nature.

**Figure 6:**
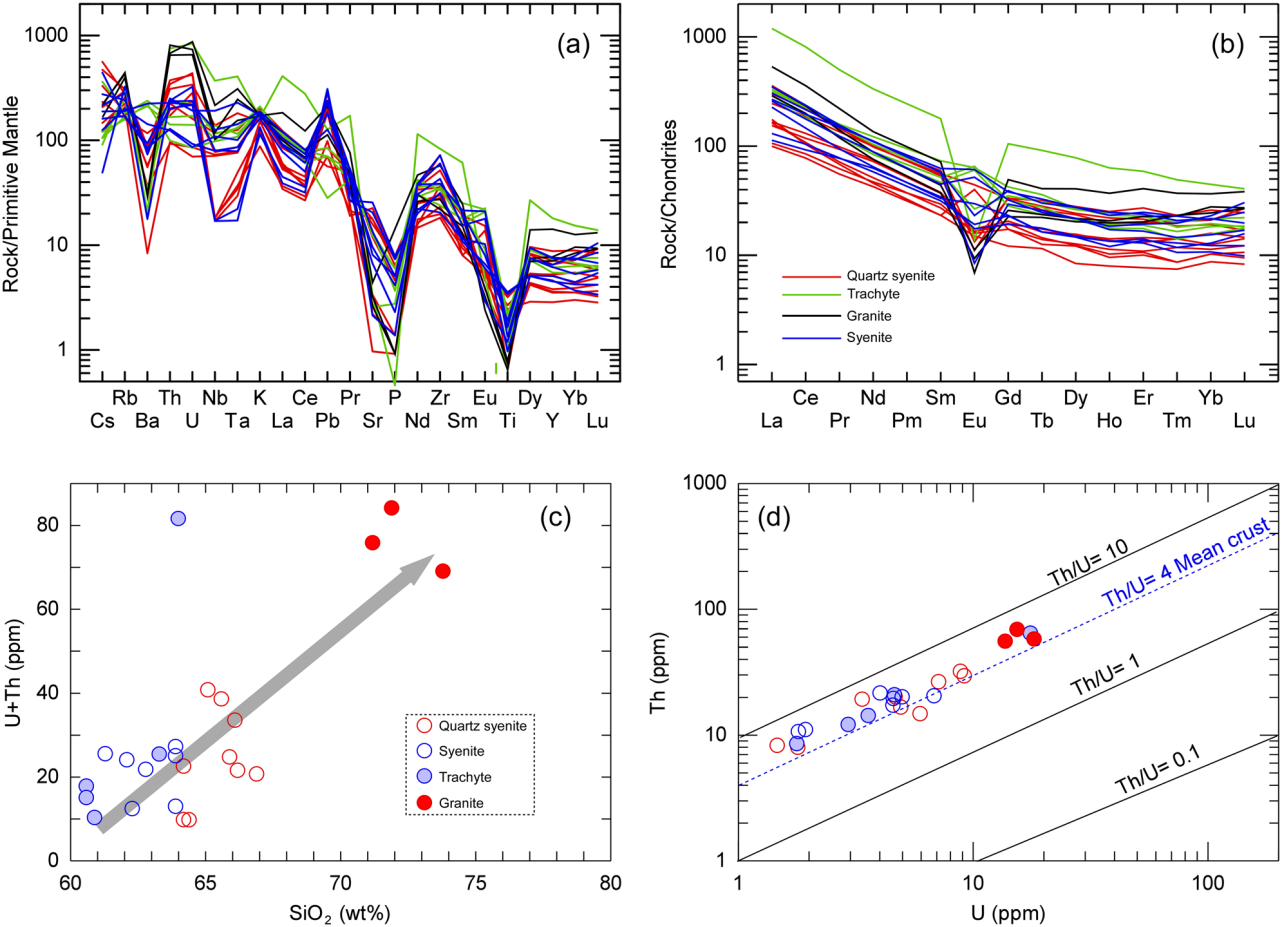
(**a**) Multi-element spider diagrams normalized to primitive mantle; (**b**) REEs patterns normalized to chondrite for the WDRC rocks^40^; (**c**) binary plot between SiO_2_ vs. U + Th; and (**d**) Th vs. U diagram of the studied WDRC rocks.

The normalized trace-element patterns of these rocks (Fig. 6a), indicate that the samples are enriched in incompatible elements with negative anomalies for Sr, Ba, P and Ti and positive anomalies for Rb, U, Th and K. This suggest that the WDRC are highly evolved rocks. Concentrations of REEs of the studied rocks are presented in Supplementary Table S2 and illustrated in chondrite normalized REE pattern (Fig. 6b)^40^. The samples have a general enrichment in LREE compared to HREE [(La/Yb)_N_ = 8.3–25.3] with clear negative Eu anomalies.

It is important to mention that the high REEs, U and Th concentrations were recorded in some Egyptian natural resources including highly fractionated rare metal granites and pegmatites^17^. The content of U and Th increased with increasing the SiO_2_ from quartz syenite to granite (Fig. 6c). Where the granites contain the highest concentration of U (14–18 ppm) and Th (55–69 ppm). The WDRC rocks have 1.5 to 18.3 ppm U and Th/U ratios of 2.5 to 5.8 (Table S1). Compared to the bulk earth ratio (Th/U = 3.9 ± 0.1)^41^, the relatively high Th/U ratios of many of the samples (Fig. 6d) from the WDRC indicate that these rocks lost significant U content during magmatic-hydrothermal fluid exsolution evolution stage. It is important to note that U and Th behaves as incompatible elements due to their large ionic radii and charge, and thus tend to concentrate in the residual molten material during the crystallization of magma^42^. This support the enrichment of the studied rocks, especially granitic samples, with these radioactive elements, as they are typically formed from residual magma^25,43^. Moreover, alkaline rocks are generally contained accessory minerals like zircon, monazite, apatite, rutile, allanite and xenotime, which can incorporate large amounts of U and Th into their crystal structures^44,45^. These minerals are relatively small in volume but can contribute significantly to the overall radioactivity of the rock.

### Radioisotope activity concentrations

**Figure 7:**
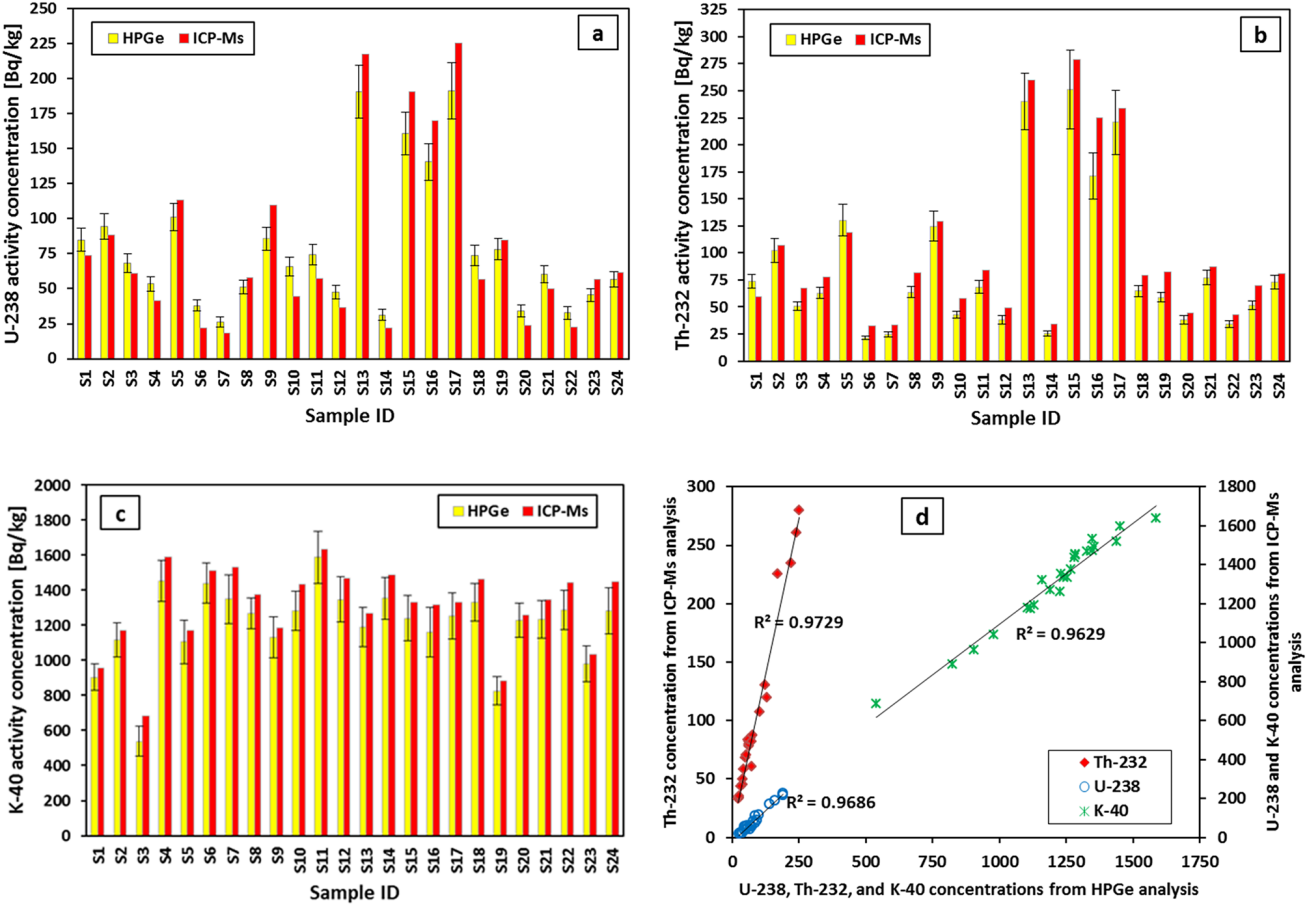
Natural radioisotope concentration values from ICP-Ms and HPGe analyses, as well as the correlation between the two techniques.

Transitioning from the geochemical characteristics to the specific radioisotope activity concentrations, the concentration of the radioisotopes (^232^Th, ^238^U(^226^Ra), and ^40^K) (Bq/kg) were determined experimentally using the HPGe detector and ICP-MS is demonstrated in Table 1. The results include the associated uncertainty, average, and Clarke values. The activity concentrations accomplished using the HPGe detector technique were utilized for assessing the health hazards of the studied rocks, whereas those obtained via the ICP-MS procedure were used to validate the results and calculate the associated radiogenic heat production for the rocks under investigation. Evidently, the radionuclide activity concentrations measured using the HPGe detector are reasonably consistent with those determined via the ICP-MS (Table 1 and Fig. 7a–c). Pearson correlation coefficients for the concentration values of ^232^Th, ^238^U, and ^40^K are 0.986, 0.984, and 0.981, respectively, indicating high levels of consistency between the two measuring techniques (Fig. 7d).

**Table 1:** Natural radioisotopes activity concentrations in the rocks of the WDRC.

Rock type	Sample ID	Analysis with HPGe detector			Clarke value CV	Analysis with ICP-Ms			Clarke value CV
		^238^U	^232^Th	^40^K		^238^U	^232^Th	^40^K	
Quartz Syenite	S1	84.92 ± 8.18	74.01 ± 6.40	904.12 ± 76.71	0.87	73.73	59.63	956.39	0.81
	S2	94.40 ± 9.06	102.24 ± 10.80	1118.16 ± 98.19	1.08	88.30	107.04	1169.78	1.21
	S3	68.55 ± 6.69	50.73 ± 4.10	538.38 ± 86.82	0.74	61.01	67.36	681.30	1.10
	S4	53.46 ± 5.30	62.90 ± 5.06	1452.22 ± 116.19	1.18	41.74	77.74	1591.41	1.86
	S5	101.44 ± 9.74	130.31 ± 14.86	1105.18 ± 124.76	1.28	113.62	119.25	1172.35	1.05
	S6	38.11 ± 3.89	21.48 ± 1.91	1439.4 ± 114.05	0.56	22.23	32.19	1511.71	1.45
	S7	26.07 ± 3.67	24.66 ± 2.31	1349.49 ± 139.94	0.95	18.15	33.41	1529.71	1.84
	S8	51.18 ± 5.10	63.66 ± 5.15	1266.68 ± 87.12	1.24	57.67	81.40	1372.88	1.41
	S9	85.82 ± 8.28	124.59 ± 13.95	1129.40 ± 117.27	1.45	109.54	129.43	1185.20	1.18
Trachyte	S10	65.82 ± 6.43	42.89 ± 3.26	1282.74 ± 112.77	0.65	44.34	57.59	1432.02	1.30
	S11	74.25 ± 7.20	68.48 ± 6.00	1587.37 ± 150.72	0.92	57.30	84.25	1632.55	1.47
	S12	47.68 ± 4.78	38.18 ± 4.05	1346.76 ± 129.36	0.80	36.43	49.04	1468.01	1.35
	S13	190.59 ± 18.82	240.08 ± 26.40	1191.57 ± 112.55	1.26	217.36	260.07	1267.48	1.20
	S14	31.41 ± 4.23	25.71 ± 2.45	1354.34 ± 120.14	0.82	21.98	34.47	1488.58	1.57
Granite	S15	160.73 ± 15.15	251.17 ± 36.50	1239.69 ± 128.33	1.56	190.81	279.20	1331.75	1.46
	S16	140.61 ± 13.29	171.14 ± 21.61	1160.89 ± 139.89	1.22	169.81	224.66	1316.32	1.32
	S17	191.39 ± 20.12	220.80 ± 29.82	1252.75 ± 130.27	1.15	225.39	234.03	1329.18	1.04
Syenite	S18	73.85 ± 7.22	64.62 ± 5.26	1329.87 ± 107.62	0.88	56.69	78.96	1462.87	1.39
	S19	78.23 ± 7.58	58.97 ± 4.59	824.81 ± 80.42	0.75	84.47	82.62	884.40	0.98
	S20	34.26 ± 4.46	37.82 ± 3.90	1228.48 ± 99.17	1.10	23.96	44.57	1257.19	1.86
	S21	60.31 ± 5.93	77.23 ± 6.89	1231.83 ± 107.52	1.28	49.77	87.10	1347.17	1.75
	S22	32.64 ± 4.56	34.10 ± 3.52	1286.23 ± 111.13	1.04	22.35	42.94	1444.87	1.92
	S23	45.43 ± 4.58	51.71 ± 4.15	980.00 ± 101.46	1.14	56.44	69.80	1033.52	1.24
	S24	56.82 ± 5.61	72.88 ± 6.21	1283.89 ± 130.83	1.28	61.75	81.20	1450.01	1.31
Overall Mean ± SE		78.67 ± 9.70	87.93 ± 13.87	1203.51 ± 45.12	1.05 ± 0.05	79.37 ± 12.61	100.75 ± 14.90	1304.86 ± 47.54	1.38 ± 0.06
WAV in regular soil^4^		35	30	400	–	35	30	400	–
WAV in building materials^48^		50	50	500	–	50	50	500	–

Based on the HPGe detector results (Table 1), the concentrations of the considered radionuclides oscillated from 26.07 ± 3.67 Bq/kg in quartz syenite (S7) to 191.39 ± 20.12 Bq/kg in granite (S17), 21.48 ± 1.91 Bq/kg in quartz syenite (S6) to 251.17 ± 36.5 Bq/kg in granite (S15) and 538.38 ± 86.82 Bq/kg in quartz syenite (S3) to 1587.37 ± 150.72 Bq/kg in trachyte (S11) for ^238^U, ^232^Th, and ^40^K, respectively. Similarly, the variation in the radioactivity level among the samples can be observed through the ICP-MS results. This variation exists even between samples of the same rock type, which might be related to the processes by which the rocks had been subjected to^34^. Additionally, the ^40^K content in all samples is the highest when compared to those of both radioisotopes ^238^U and ^232^Th. In general, the high concentration of ^40^K in all the samples is connected to the enrichment of the potash feldspar mineral in all the rocks under investigation^46^. The computations have disclosed that the majority of the studied samples had a Clarke value larger than one (Table 1), reflecting Th-enrichment^47^.

**Figure 8:**
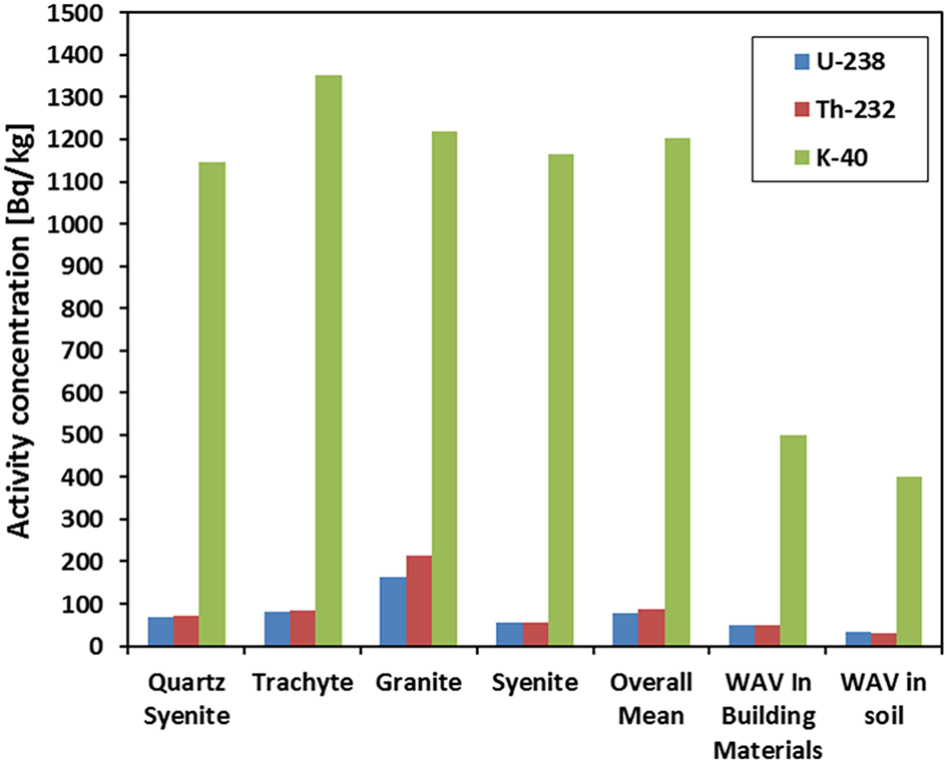
The mean values of the radioisotopes in WDRC rocks compared to their corresponding WAVs in regular soil and building materials.

Touching on the average values of ^238^U, ^40^K, and ^232^Th activity concentrations in the investigated rocks of WDRC, the results designated that granite had the highest average concentrations of both ^238^U (^226^Ra) and ^232^Th, with values of 164.24 ± 14.76 and 214.37 ± 23.33 Bq/kg, respectively, while trachyte had the highest average concentration of ^40^K, with a value of 1352.56 ± 65.56 Bq/kg (Fig. 8). Conversely, syenite had the lowest mean concentrations of ^238^U and ^232^Th, represented by 54.51 ± 6.81 and 56.76 ± 6.25 Bq/kg, respectively, whereas quartz syenite had the lowest mean concentration of ^40^K, estimated at 1144.78 ± 96.19 Bq/kg (Fig. 8). Clearly, the average concentration of the radioisotopes of interest is all higher than their worldwide average values (WAVs) in typical soils (35, 30, and 400 Bq/kg for ^238^U(^226^Ra), ^232^Th, and ^40^K, respectively, as in UNSCEAR^4^) and in building materials (50, 50, and 500 Bq/kg for ^238^U (^226^Ra), ^232^Th, and ^40^K, respectively, as in UNSCEAR^48^). So basically, the overall average values of the terrestrial radionuclide concentrations exceeded the WAVs (Table 1), requiring knowledge of the potential radiation exposure not only in the area being studied but also in places where these types of rocks might be used. Table 2 summarizes the concentrations of ^238^U, ^232^Th, and ^40^K in the rocks examined herein, compared to some previous studies conducted in Egypt and other countries. The results stipulated that the radioisotope concentration values for all rocks from the WDRC fall within the range of corresponding rocks in published data.

**Table 2:** The concentrations of radioactive isotopes compared to previous studies.

Country name	Rock type	Activity concentration (Bq/kg)			References
		^226^Ra	^232^Th	^40^K	
Egypt (Dib)	Quartz Syenite	67.11	72.73	1144.78	Present work
Egypt (Dib)	Trachyte	81.95	83.07	1352.56	Present work
Egypt (Dib)	Granite	164.24	214.37	1217.78	Present work
Egypt (Dib)	Syenite	54.51	56.76	1166.44	Present work
Egypt (Seih-Sidri)	Older Granites	32.11	28.08	557.46	^63^
Egypt	Commercial Granite	66.6	102.98	1063.15	^64^
Nigeria (Ondo and Ekiti states)	Metamorphic	13.38	3.31	4.75	^65^
Serbia	Phosphate	693	18	102	^66^
Pakistan (Ambela)	Granite	659	598	1218	^67^
Brazil	Syenite	600	310	2300	^68^
Turkey (Sandıklı-Suhut)	Trachyte	455.72	332.19	1994.46	^69^
China	Commercial Granite	355.9	317.9	1636.5	^70^
Italy (Sicily)	Basalt	58.6	40.7	498	^71^
USA	Commercial granites	31	61	1210	^72^
South Africa	Quartz Syenite	148.08	183.15	1022.01	^73^
South Africa	Trachyte	143.14	63.1	2415.66	^73^
South Africa	Granite	74.04	84.57	1052.98	^73^
South Africa	Syenite	150.55	168.5	1022.01	^73^
Saudi Arabia	Decorative granite	54.5	43.4	677.7	^46^
India	Granite	82	112	1908	^74^

### Radiogenic heat production (RHP) evaluation

**Figure 9:**
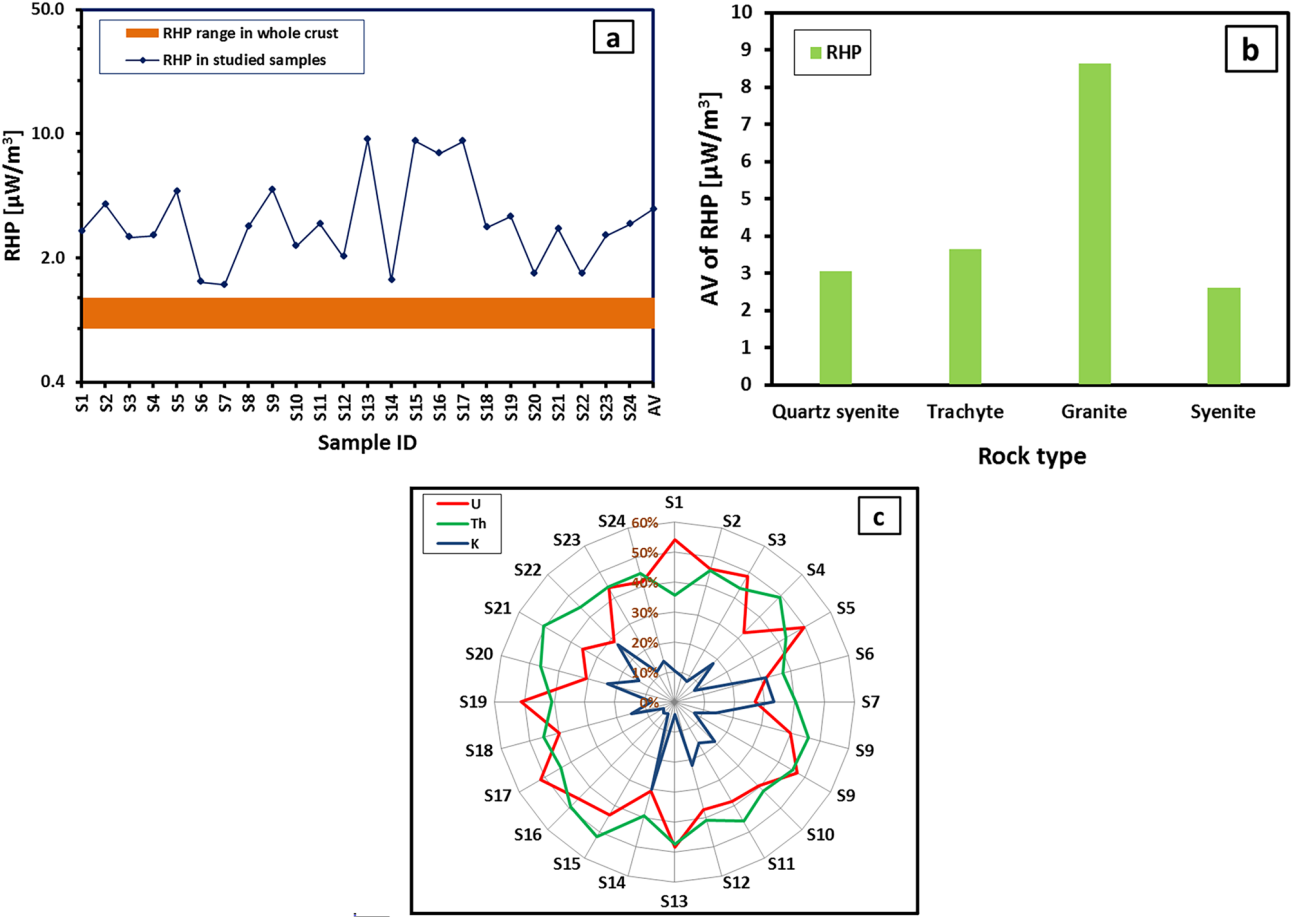
Variations of the radioactive heat production (RHP) values and differences in the contribution of each radioelement to these values for WDRC rock types samples.

During the radioactive disintegration of the radionuclides within rocks, energy is released, with a large portion of energy transformed into heat. The contributions of the ^238^U, ^232^Th, and ^40^K decay chains to this thermal energy are significant compared to the other radioisotopes. The heat produced in a second from a volume of rock through radioactive disintegration is known as radiogenic heat production (RHP). The latter is influenced by the geochemical characteristics of the rock and can be estimated as^49,50^:

4 $$RHP\left[\mathrm{\mu W }{{\text{m}}}^{-3}\right]=\left({9.52\mathrm{ C}}_{{\text{U}}}+{ 2.56\mathrm{ C}}_{{\text{Th}}}+{3.48\mathrm{ C}}_{{\text{K}}}\right)\uprho /{10}^{5}$$

where ρ is the density of the studied rocks, taken as 2700 kg/m^3^, C_U_ and C_Th_ denote uranium and thorium concentrations, respectively, measured in parts per million (ppm), and C_K_ characterizes the potassium concentration measured in percent (%). Studying the RHP forms a crucial part of our study, connecting the elemental concentrations of U, Th, and K to their heat-producing capabilities. Depending on the measured elemental concentrations of U, Th, and K in WDRC, the RHP (µWm^−3^) was computed (Table 3 and Fig. 9). The values of all four rock types were higher than those of the Earth's crust (0.8–1.2 μWm^−3^^51,52^) (Table 3 and Fig. 9a). They vary from 1.41 to 4.84 μWm^−3^ in quartz syenite (avg. = 3.07 μWm^−3^), from 1.49 to 9.33 (avg. = 3.66 μWm^−3^) in trachyte, from 7.75 to 9.12 μWm^-3^ (avg. = 8.64 μWm^−3^) in granite, and from 1.63 to 3.43 μWm^−3^ (avg. = 2.62 μWm^−3^) in syenite. Accordingly, the highest mean value of RHP is for the granite samples, whereas the lowest mean is for the syenite samples (Fig. 9b).

**Table 3:** Elemental concentrations of the radioelements (U, Th and K) and their contributions to the associated radiogenic heat production in WDRC rocks.

Rock type	Sample ID	Radioelement concentrations from ICP-Ms				Heat production rate [μW/m3] due radioelement			Total RHP [µW/m3]	Contribution [%] of radioelement in total RHP		
		U [ppm]	Th [ppm]	K [%]	U		Th	K		U	Th	K
Quartz syenite	S1	5.97	14.65	3.09	1.53		1.01	0.29	2.84	54.08	35.69	10.23
	S2	7.15	26.30	3.78	1.84		1.82	0.35	4.01	45.82	45.33	8.85
	S3	4.94	16.55	2.20	1.27		1.14	0.21	2.62	48.46	43.65	7.89
	S4	3.38	19.10	5.14	0.87		1.32	0.48	2.67	32.52	49.41	18.07
	S5	9.20	29.30	3.79	2.36		2.03	0.36	4.75	49.83	42.68	7.49
	S6	1.80	7.91	4.88	0.46		0.55	0.46	1.47	31.52	37.24	31.24
	S7	1.47	8.21	4.94	0.38		0.57	0.46	1.41	26.81	40.26	32.93
	S9	4.67	20.00	4.43	1.20		1.38	0.42	3.00	40.02	46.09	13.89
	S9	8.87	31.80	3.83	2.28		2.20	0.36	4.84	47.13	45.44	7.43
Trachyte	S10	3.59	14.15	4.62	0.92		0.98	0.43	2.34	39.51	41.88	18.60
	S11	4.64	20.70	5.27	1.19		1.43	0.50	3.12	38.24	45.88	15.88
	S12	2.95	12.05	4.74	0.76		0.83	0.45	2.04	37.23	40.90	21.87
	S13	17.60	63.90	4.09	4.52		4.42	0.38	9.33	48.51	47.36	4.12
	S14	1.78	8.47	4.81	0.46		0.59	0.45	1.49	30.61	39.17	30.22
Granite	S15	15.45	68.60	4.30	3.97		4.74	0.40	9.12	43.56	52.01	4.43
	S16	13.75	55.20	4.25	3.53		3.82	0.40	7.75	45.61	49.24	5.15
	S17	18.25	57.50	4.29	4.69		3.97	0.40	9.07	51.73	43.83	4.45
Syenite	S18	4.59	19.40	4.72	1.18		1.34	0.44	2.96	39.80	45.23	14.97
	S19	6.84	20.30	2.86	1.76		1.40	0.27	3.43	51.26	40.91	7.82
	S20	1.94	10.95	4.06	0.50		0.76	0.38	1.64	30.46	46.24	23.30
	S21	4.03	21.40	4.35	1.04		1.48	0.41	2.92	35.43	50.59	13.98
	S22	1.81	10.55	4.67	0.47		0.73	0.44	1.63	28.49	44.66	26.85
	S23	4.57	17.15	3.34	1.17		1.19	0.31	2.67	43.94	44.34	11.73
	S24	5.00	19.95	4.68	1.29		1.38	0.44	3.10	41.40	44.42	14.17
Average		6.43	24.75	4.21	1.65		1.71	0.40	3.76	40.92	44.27	14.82

The percentage contributions of the radioelements U, Th, and K to the RHP level of the samples examined are illustrated in Table 3 and Fig. 9c. Thorium contributed the most to the majority of the samples, with an average of 44.27% of the total, followed by uranium with a close ratio of 40.92% and potassium with a smaller value of 14.27% (Fig. 9c and Table 3). The results specified that, on average, these three radionuclides contributed, respectively, 41.80%, 42.87%, and 15.34% in the RHP for quartz syenite samples; 38.82%, 43.04%, and 18.14% for trachyte samples; 46.97%, 48.36%, and 4.68% for granite samples; and 38.68%, 45.20%, and 16.12% for syenite samples. It appears that U and Th provided nearly similar ratios to the radiogenic heat production in most samples, whereas K contributed a smaller quantity. This means that U and Th decay more slowly and release heat over longer period of time, contributing more to the overall radiogenic heat production; K decays rapidly and contributes less to the overall heat production. These findings not only highlight the significant role of U and Th in long-term heat production but also align with their geochemical behavior and concentration patterns.

### Radiological effects assessment

**Figure 10:**
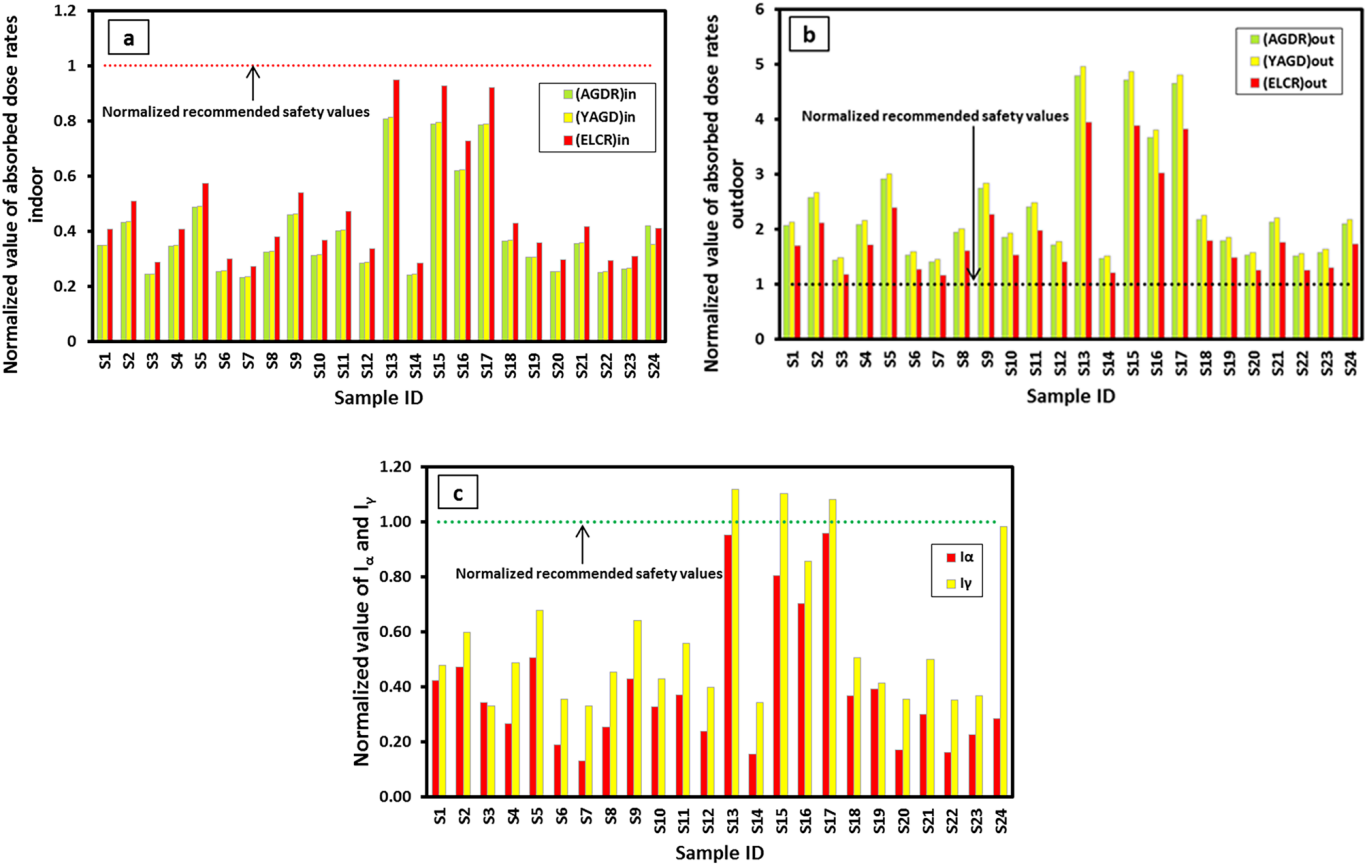
Normalization of radiological parameter values with their recommended safety values for comparison purposes.

Assessing the radiological effects of WDRC rocks is critical to understanding their practical implications. The potential radiation risks from WDRC rocks, due to the presence of the radioisotopes ^238^U, ^232^Th, and ^40^K, were assessed by calculating key radiological hazard parameters. Table 4 shows the values of computed radiological parameters for the examined samples, while Fig. 10 compares these values to safety limits, which have been normalized according to recommended standards. The calculated parameters herein are:

**Table 4:** The radiological risk parameters for WDRC rock samples.

Rock type	Sample ID	(AGDR)_in_ [nGy/h]	(AGDR)_out_ [nGy/h]	(YAGD)_in_ [mSv/y]	(YAGD)_out_ [mSv/y]	(YAGD)_tot_ [mSv/y]	(ELCR)_in_ × 10^−3^	(ELCR)_out_ × 10^−3^	(ELCR)_tot_ × 10^−3^	I_α_	I_γ_
Quartz Syenite	S1	29.23	121.64	0.14	0.15	0.29	0.47	0.49	0.97	0.42	0.95
	S2	36.38	151.99	0.18	0.19	0.37	0.59	0.62	1.20	0.47	1.20
	S3	20.50	84.76	0.10	0.10	0.20	0.33	0.34	0.68	0.34	0.66
	S4	29.16	123.25	0.14	0.15	0.29	0.47	0.50	0.97	0.27	0.98
	S5	41.03	171.66	0.20	0.21	0.41	0.66	0.70	1.36	0.51	1.36
	S6	21.40	90.60	0.11	0.11	0.22	0.35	0.37	0.71	0.19	0.71
	S7	19.54	83.21	0.10	0.10	0.20	0.32	0.34	0.65	0.13	0.66
	S8	27.21	114.92	0.13	0.14	0.27	0.44	0.47	0.91	0.26	0.91
	S9	38.58	162.00	0.19	0.20	0.39	0.63	0.66	1.28	0.43	1.29
Trachyte	S10	26.22	109.80	0.13	0.13	0.26	0.42	0.44	0.87	0.33	0.86
	S11	33.74	141.86	0.17	0.17	0.34	0.55	0.57	1.12	0.37	1.12
	S12	24.00	101.25	0.12	0.12	0.24	0.39	0.41	0.80	0.24	0.80
	S13	67.92	282.75	0.33	0.35	0.68	1.10	1.15	2.25	0.95	2.23
	S14	20.37	86.52	0.10	0.11	0.21	0.33	0.35	0.68	0.16	0.68
Granite	S15	66.35	277.66	0.33	0.34	0.67	1.07	1.12	2.20	0.80	2.20
	S16	51.98	216.74	0.26	0.27	0.52	0.84	0.88	1.72	0.70	1.71
	S17	65.91	274.03	0.32	0.34	0.66	1.07	1.11	2.18	0.96	2.16
Syenite	S18	30.68	128.60	0.15	0.16	0.31	0.50	0.52	1.02	0.37	1.01
	S19	25.56	106.15	0.13	0.13	0.26	0.41	0.43	0.84	0.39	0.83
	S20	21.20	89.90	0.10	0.11	0.21	0.34	0.36	0.71	0.17	0.71
	S21	29.87	125.88	0.15	0.15	0.30	0.48	0.51	0.99	0.30	1.00
	S22	21.04	89.31	0.10	0.11	0.21	0.34	0.36	0.70	0.16	0.71
	S23	22.10	93.09	0.11	0.11	0.22	0.36	0.38	0.73	0.23	0.74
	S24	29.35	123.81	0.14	0.15	0.30	0.48	0.50	0.98	0.28	0.98
Overall AV		33.30	139.64	0.16	0.17	0.33	0.54	0.57	1.11	0.39	1.10
Recommended safety limit (RSL)		84^a^	59^a^	0.41^a^	0.07^a^	0.48^a^	1.16^b^	0.29^c^	1.45^b,c^	1f.	2^ g^

RSL reported in:

^a^UNSCEAR^4^.

^b^Sidique, et al.^38^.

^c^Qureshi, et al.^56^.

^d^Beretka and Matthew^75^.

^e^Krieger^76^.

^f^ICRP^60^.

^g^European Commission^53^.

#### Absorbed gamma dose rate (AGDR)

The indoor and outdoor absorbed gamma dose rates (AGDR_in_ and AGDR_out_) in nGy/h for the γ-radiation energy in the air were estimated using Eqs. (5a) and (5b) suggested by the European Commission^53^ and UNSCEAR^4^, respectively.

5a $${{\text{AGDR}}}_{in}\left[{\text{nGy}}/{\text{h}}\right]={0.12\times {\text{AC}}}_{{\text{Ra}}}+{ 0.14\times {\text{AC}}}_{{\text{Th}}}+{0.0096\times {\text{AC}}}_{{\text{K}}}$$

5b $${{\text{AGDR}}}_{out}\left[{\text{nGy}}/\mathrm{ h}\right]={0.462\times {\text{AC}}}_{{\text{Ra}}}+{ 0.604\times {\text{AC}}}_{{\text{Th}}}+{0.0417\times {\text{AC}}}_{{\text{K}}}$$

where AC_K_, AC_Th_, and AC_U_ denote the activity concentrations of the radioisotopes ^40^K, ^232^Th, and ^238^U(^226^Ra), respectively, measured in Bq/kg. Notably, Eq. (5a) proposed by the European Commission^53^ can be applied to evaluate the indoor gamma dose rates (AGDR_in_) incurred by the population from rocks, such as those studied, when used as decorative materials in rooms of buildings. Meanwhile, Eq. (5b) announced by UNSCEAR^4^ can be utilized to evaluate the outdoor gamma dose rates (AGDR_out_) received by workers in the area under investigation. The indoor absorbed gamma dose rate (AGDR_in_) values (Table 4) varied from 19.54 nGy/h in S7 (quartz syenite) to 67.92 nGy/h in S13 (trachyte) (mean 33.3 nGy/h). Accordingly, none of the values of AGDR_in_ in the investigated samples surpassed the recommended safety limits (RSL) of 70 nGy/h^53^ and 84 nGy/h^4^ (Fig. 10a). As such, none of the rocks examined herein are likely to exhibit substantial radiological hazards to people when used as surface-building materials. Differently, the outdoor absorbed dose rates (AGDRout), calculated from Eq. (5b) and applied to workers in the area under investigation, exceeded the recommended safety limit (RSL) of 59 nGy/h^4^ (Fig. 10b), as they spanned the range from 83.21 nGy/h in S7 (quartz syenite) to 282.75 nGy/h in S13 (trachyte) (mean 139.64) nGy/h (Table 4). As such, if proper safety measures are not followed, the WDRC rocks may pose a radiological hazard to workers.

#### Yearly effective gamma dose (YEGD)

Depending on the AGDR values, the indoor, outdoor, and total yearly effective gamma dose (YEGD_in_, YEGD_out_, and YEGD_tot_) rates (mSv/y) were calculated using Eqs. (6a), (6b), and (6c), respectively, as reported previously by^4^.

6a $${{\text{YEGD}}}_{{\varvec{i}}{\varvec{n}}}\left[{{\text{mSvy}}}^{-1}\right]={{\text{AGDR}}}_{in}\left[\mathrm{nGy }{{\text{h}}}^{-1}\right]\times 8766\times 0.8\times 0.7{{\text{SvGy}}}^{-1}\times {10}^{-6}$$

6b $${{\text{YEGD}}}_{out}\left[{{\text{mSvy}}}^{-1}\right]={{\text{AGDR}}}_{out}\left[\mathrm{nGy }{{\text{h}}}^{-1}\right]\times 8766\times 0.2\times 0.7{{\text{SvGy}}}^{-1}\times {10}^{-6}$$

6c $${{\text{YEGD}}}_{tot}\left[{{\text{mSvy}}}^{-1}\right]={{\text{YEGD}}}_{in}\left[{{\text{mSvy}}}^{-1}\right]+{{\text{YEGD}}}_{out}\left[{{\text{mSvy}}}^{-1}\right]$$

where the indoor and outdoor occupancy factors are 0.8 and 0.2, respectively; the conversion factor 0.7 × 10^–6^ SvG/y is used to convert AGDR to YEGD; the yearly hours number is 8760 h. According to the European Commission^53^, materials used superficially (such as the rocks under investigation) should not be subjected to restrictions regarding radioactivity, as long as the increase in the yearly effective dose due to the excess gamma radiation from these materials, does not exceed 0.3 mSv/y (the exemption level). The benchmark to maintain radiation gamma doses is emphasized within the range of 0.3 to 1 mSv/y (control level) for the purpose of radiation protection and safety. Also, it is outlined that materials with gamma doses exceeding 1 mSv/y (the threatening level) should not be recommended for use in buildings. The YEGD_in_ values for the investigated samples (Table 4), except for S13 (trachyte), S15 (granite), and S17 (granite), aren't beyond the exemption level (0.3 mSv/y). Although those three samples have values (0.33, 0.33, and 0.32 mSv/y) slightly higher than the exemption level, they are still below: the limit dose of 1 mSv/y^53^ and the recommended safety value of 0.41 mSv/y^4^ (Fig. 10a). Also, the YEGD_in_ mean values for the examined rock types cover a span from 0.13 mSv/y in syenite samples to 0.30 mSv/y in granite samples (with an overall average value of 0.16 mSv/y), i.e. all of these mean values oscillated in the exemption level (European Commission^53^). In view of this, it is possible to use WDRC rocks as safe building materials for surface applications. In contrast, the YEGD_out_ values observed in all examined samples were found to exceed the global average of 0.07 mSv/y^4^ (Table 4 and Fig. 10b). This finding underscores the significance of adhering to appropriate safety protocols for safeguarding workers against radiation exposure during work within the WDRC area. As displayed in Table 4, while the overall mean value of YEGD_out_ (0.17 mSv/y) is about 2.43 times greater than that of the world average of 0.07^4^ mSv/y, that of the YEGD_in_ (0.16 mSv/y) is 2.56 times smaller than the world average of 0.41 mSv/y^4^. Furthermore, the YEGD_tot_ (YEGD_out_ + YEGD_in_) mean values vary remarkably among the rock types, with the lowest value of 0.20 mSvy^-1^ found in syenite and the highest value of 0.68 mSvy^-1^ observed in granite.

#### Excess lifetime cancer risk (ELCR)

The ELCR quantifies the cancer risk for an individual exposed to low levels of gamma radiation. Each of the indoor, outdoor, and total ELCRs (ELCR_in_, ELCR_in_, and ELCR_tot_) were calculated using the computed values of the yearly effective doses, as seen in Eqs. (7a), (7b), and (7c):

7a $${{\text{ELCR}}}_{out}={{\text{YEGD}}}_{out}\times {\text{MLE}}\times {\text{RF}}$$

7b $${{\text{ELCR}}}_{in}={{\text{YEGD}}}_{in}\times {\text{MLE}}\times {\text{RF}}$$

7c $${{\text{ELCR}}}_{tot}={{\text{ELCR}}}_{in}+{{\text{ELCR}}}_{out}$$

where MLE and RF are the factors determining the mean life expectancy (66 years)^54^ and the risk of fatal stochastic impact (0.05 Sv^-1^ for the overall population), respectively^55^. Considering the estimated ELCR (Table 4), all the values of ELCR_in_ were found to be below the recommended threshold of 1.16 × 10^–3^ (Sidique, et al.^38^ and Qureshi, et al.^56^) (Fig. 10a). This implies a very low potential for cancer occurrence due to gamma-ray exposure over an individual's lifetime (66 years) when the WDRC rocks are used as ornamental covering materials in buildings. On the other hand, the ELCR_out_ values were found to be above the recommended threshold of 0.29 × 10^–3^^4^ for outdoor ELCR in all investigated samples (Fig. 10b). The calculated average ELCR_in_, ELCR_out_, and ELCR_tot_ values in the rock types under investigation range from the lowest value in syenite to the highest value in granite samples, with ranges (0.42–0.99) × 10^–3^, (0.44–1.04) × 10^–3^, and (0.85–2.03) × 10^–3^, respectively, and with overall average values of 0.54 × 10^–3^, 0.57 × 10^–3^, and 1.11 × 10^–3^, respectively. This emphasizes the importance of following safety procedures to protect workers from radiation exposure to the WDRC granites.

#### Gamma and Alpha indices (I_γ_ and I_α_)

The gamma index (I_γ_) given in Eq. (8a)^53^ is applied to consider the γ-ray risk related to the natural radionuclides in WDRC rock types when served as construction materials. The European Commission^53^ proposed that I_γ_ ≤ 2, 2 ≤ I_γ_ ≤ 6, and I_γ_ > 6 be balanced to yearly effective γ doses of ≤ 0.3 (exemption level), ≤ 1 (control level), and > 1 mSv/y (threatening level), respectively.

8a $${{\text{I}}}_{\upgamma }=\frac{{{\text{AC}}}_{{\text{Ra}}}}{300{{\text{Bqkg}}}^{-1}}+\frac{{{\text{AC}}}_{{\text{Th}}}}{200{{\text{Bqkg}}}^{-1}}+\frac{{{\text{AC}}}_{{\text{K}}}}{3000{{\text{Bqkg}}}^{-1}}$$

8b $${{\text{I}}}_{\mathrm{\alpha }}=\frac{{{\text{AC}}}_{{\text{Ra}}}}{200\mathrm{ Bq}{{\text{kg}}}^{-1}}$$

Furthermore, to quantify the internal exposure levels to excessive α-radiation from inhaling radon gas, from the rocks used as building materials, the alpha index (I_α_) was applied. This index was estimated through Eq. (8b)^57^, involving ^226^R activity concentration (AC_Ra_) in Bq/kg. It is noted that if the AC_Ra_ in a building material exceeds 200 Bq/kg (I_α_ > 1), it may result in indoor radon (Rn) concentrations surpassing the allowable level of 200 Bq/m^3^. Conversely, if the AC_Ra_ is below 100 Bq/kg (Iα < 0.5), indoor Rn concentrations exceeding 200 Bq/m^3^ are unlikely. The Radiation Protection Authorities in the Nordic countries recommended 100 Bq/kg as the exemption level (I_α_ = 0.5) and 200 Bq/kg as the upper level (I_α_ = 1) for AC_Ra_ in building materials^34,58^. Accordingly, the I_α_ should be less than one in order to maintain indoor Rn concentrations below the allowable level of 200 Bq/m^3^.

The I_γ_ values for the studied rock samples (Table 4 and Fig. 10c), except for S13 (trachyte), S15 (granite), and S17 (granite) are below the exemption limit of Iγ < 2, which corresponds to a dose less than 0.3 mSv/y. Even though the samples S13, S15, and S17 have I_γ_ values (2.23, 2.20, and 2.16) slightly higher than the exemption level (I_γ_ = 2), they are still below the threatening limit (I_γ_ = 6) that corresponds to the limit dose of 1 mSv/y. Arguably, this is the same finding as what we have obtained through the annual indoor gamma dose calculations (YEGD_in_) discussed above. The calculations revealed that the mean values of I_γ_ for all examined rocks do not exceed 2 (exemption level), except for granite, which has an average value of 2.03, touching approximately the exemption limit. Thus, the WDRC rocks, including granite, are deemed suitable for unrestricted use as surface construction materials.

The I_α_ values in the considered samples vary between 0.13 and 0.96, with an overall average value of 0.39 (Table 4). Accordingly, across all the different types of rocks that were studied, none of their average I_α_ values were greater than 1 (Fig. 10c). The quartz syenite, trachyte, and syenite are within the exemption level (I_α_ = 0.5) for building materials in terms of Rn exposure, with mean I_α_ values of 0.34, 0.41, and 0.27, respectively. On the other hand, granite is located below the upper recommended limit (I_α_ = 1) for Rn exposure, with a mean I_α_ value of 0.82. Accordingly, the rock types studied fall within the range of the safe indoor radon exposure levels recommended for buildings (European Commission^59^, ICRP^60^, and Nordic^58^).

The integration of radiological parameters with the previously discussed geochemical and radiogenic properties provides a comprehensive understanding of the potential impacts and applications of the WDRC rocks.

### Multivariate statistical investigations

**Figure 11:**
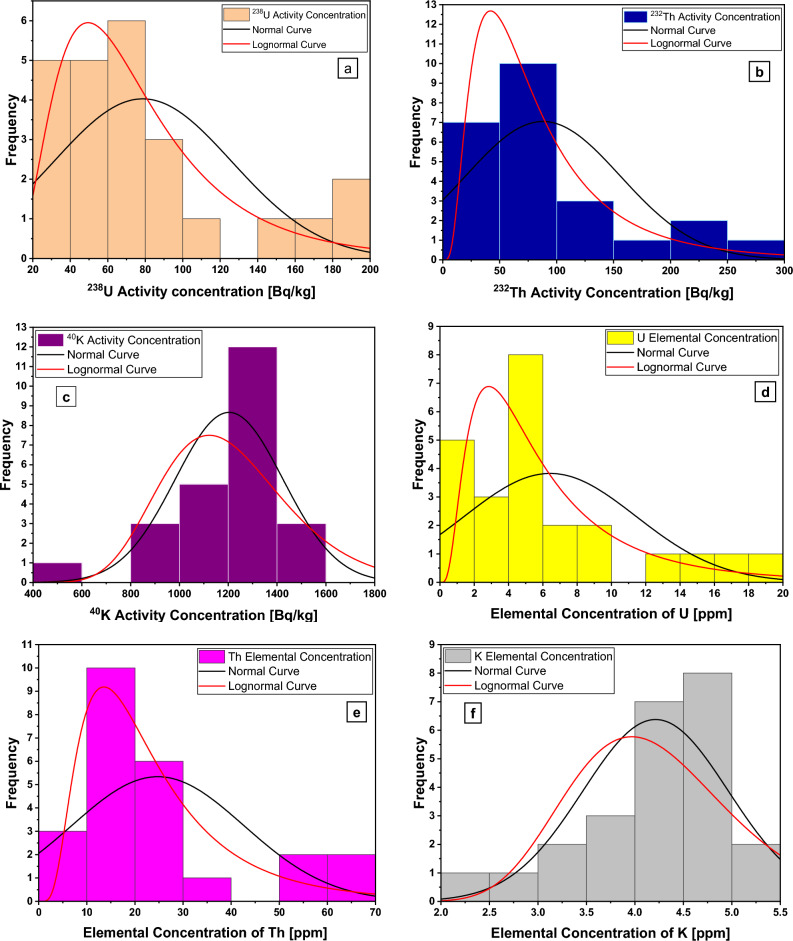
Distribution curves for the activity and elemental concentration values of radionuclides.

Multivariate statistical analysis is essential to enhance our comprehensive study by understanding the relationships between the natural radionuclides and related radiological parameters. The fundamental statistical measures pertaining to the natural radionuclides and the related radiological parameters are shown in Supplementary Table S3. Obviously, all variable standard deviation values are smaller than the mean values, suggesting that data points are relatively close to the mean, reflecting a higher level of uniformity. Furthermore, the non-zero skewness values observed for all variables (Supplementary Table S3) indicated the existence of distributions that deviate from symmetry (Fig. 11). A positive skewness value designated the distribution as having a longer tail on the right side and being skewed to the right, while a negative skewness value pointed to a longer tail on the left side and a skew to the left (Fig. 11). Skewness for all variables, except the values of K concentration and its own radioisotope ^40^K concentration, have positive values (Supplementary Table S3). Additionally, all variables exhibit positive kurtosis values (Supplementary Table S3), implying a leptokurtic distribution as the distribution curves of these variables are more peaked than the standard normal curve (Fig. 11).

In fact, the distribution normality examination was accomplished through the utilization of both the modified Kolmogorov–Smirnov (KS) test and the Lilliefors test. These tests rely on the p-value, which assesses the probability of the null hypothesis. Failure to reject the null hypothesis occurs when the p-value > 0.05 suggests a normal distribution. Conversely, if the p-value is < 0.05, the null hypothesis is rejected, indicating the presence of an asymmetrical distribution (Supplementary Table S3). Moreover, the analysis undertaken to compare the goodness of fit tests for normal and log-normal distributions revealed that the datasets concerning ^40^K activity concentration and K elemental concentration manifested a statistically significant conformity to the normal distribution, while the remaining variables displayed a statistically significant conformity to the log-normal distribution (Supplementary Table S3 and Fig. 11).

**Figure 12:**
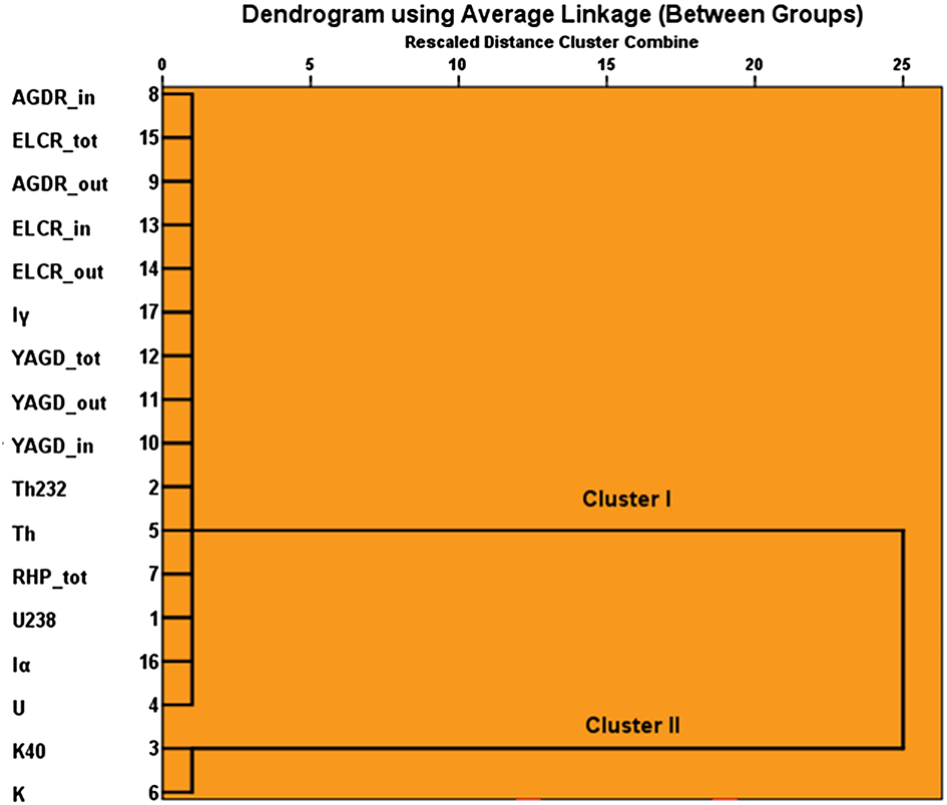
The dendrogram derived from the HCA illustrating the relationship among the considered variables.

In an effort to show the impact of natural radionuclides on the levels of natural radioactivity present in the rocks under investigation, through the examination of the relation between these nuclides and the radiological parameters, a hierarchical cluster analysis (HCA) was conducted. The analysis employed the single linkage method in conjunction with the correlation coefficient distance between the variables. The analysis outputs are represented in the Pearson correlation matrix of variables (Supplementary Table S4), as well as the dendrogram (Fig. 12). The dendrogram revealed an interesting correlation between radiological parameters and radioactive nuclides, as all 17 parameters were categorized into two significant clusters based on similarities. Cluster I, which involve all radiological parameters as well as ^238^U, ^232^Th, U, and Th, signifying radioactivity in the studied rocks, is mainly tied to U and Th concentrations. Cluster II just encompasses ^40^K and K, suggesting that K concentration in WDRC rocks minimally impacts radiation hazards. This is aligned with the Pearson correlation analysis (Supplementary Table S4), as one can observe the high degree of correlation among all radiological parameters, as well as between these parameters and uranium and thorium. In contrast, an extremely low degree of correlation can be observed between these parameters and potassium.

### Conclusion remarks

1. The WDRC, the oldest in the Egyptian basement complex at approximately 578 ± 16 Ma, resides in the north Eastern Desert. It composed essentially of syenites, quartz syenites, trachytes, and granite, and characterized by varying mineral compositions and textures, including K-feldspar, plagioclase, alkali-amphibole, pyroxene, and accessory minerals like zircon and allanite.
2. Geochemically, The WDRC samples show high concentrations of SiO_2_, Al_2_O_3_, and total alkalis alongside variable CaO and Fe_2_O_3_. They enriched with incompatible elements with positive anomalies for Rb, U, Th, and K, suggesting high evolution. U and Th concentrations increase with SiO_2_, with granites displaying the highest U and Th levels. The occurrence of zircon, allanite and monazite contribute enhance the enrichment of the studied rocks with high radioactive elements.
3. The HPGe detector and ICP-MS measurements demonstrated consistency, supported by high Pearson correlation coefficients. Samples exhibited varying radioactivity levels, with granite displaying the highest ^238^U and ^232^Th concentrations and trachyte showing the highest 40 K content. These concentrations exceeded worldwide average values (WAVs) in soils and building materials. The study emphasized the need for assessing potential radiation exposure, considering the usage of such rocks beyond the study area. Furthermore, the radioisotope concentrations in WDRC rocks fell within the range reported in previous studies conducted in Egypt and other countries.
4. The computed Radiogenic Heat Production (RHP) in WDRC rocks exceeded Earth's crust values. The data revealed that uranium and thorium significantly contribute to radiogenic heat production, releasing heat over longer periods compared to potassium, which decays rapidly and contributes less to overall heat production.
5. The absorbed gamma dose rates for indoor exposure (AGDR_in_) are below the recommended safety limit (RSL). Conversely, outdoor absorbed dose rates (AGDR_out_) surpassed the RSL values. The yearly effective gamma dose (YEGD_in_) values for most samples were within the exemption level, except for three samples slightly above the limit. For outdoor exposure (YEGD_out_), all samples exceeded the global average, emphasizing the need for safety measures for workers. The estimated excess lifetime cancer risk (ELCR) for indoor exposure was low, contrasting the higher risk for outdoor exposure, especially for workers. The rocks, including granite, are considered suitable for unrestricted surface construction material use, but caution is advised for worker safety regarding outdoor exposure.
6. Besides, through the application of statistical methods, including Pearson correlation and HCA, it was established that any radiation hazard in the considered rocks can mainly be attributed to the concentrations of U and Th, whereas the contribution of K is minimal. The present research serves as the inaugural reference dataset on natural radionuclides in the area; therefore, we recommend follow-up measures to record changes and formulate a reasonable pollution control strategy. The WDRC rocks fall within the safe range of exposure levels recommended for buildings, but they might pose a radiological hazard to local workers.

### Supplementary Information

Supplementary Tables.

## Data Availability

All data generated or analyzed during this study are included in this published article and its supplementary information files.
